# Effectiveness of the Close Collaboration with Parents intervention on parent-infant closeness in NICU

**DOI:** 10.1186/s12887-020-02474-2

**Published:** 2021-01-11

**Authors:** Felix B. He, Anna Axelin, Sari Ahlqvist-Björkroth, Simo Raiskila, Eliisa Löyttyniemi, Liisa Lehtonen

**Affiliations:** 1 Department of Pediatrics and Adolescent Medicine, Turku University Hospital, Kiinamyllynkatu 4-8, 20521 Turku, Finland; 2 University of Turku, Turku, Finland; 3 Department of Nursing Science, University of Turku, Turku, Finland

**Keywords:** Parental presence, Skin-to-skin contact, Family centered care, Kangaroo care, NICU

## Abstract

**Background:**

Parent-infant closeness during hospital care of newborns has many benefits for both infants and parents. We developed an educational intervention for neonatal staff, Close Collaboration with Parents, to increase parent-infant closeness during hospital care. The aim of this study was to evaluate the effectiveness of the intervention on parent-infant closeness in nine hospitals in Finland.

**Methods:**

Parents of hospitalized infants were recruited in the hospitals during 3-month periods before and after the Close Collaboration with Parents intervention. The data were collected using daily Closeness diaries. Mothers and fathers separately filled in the time they spent in the hospital and the time of skin-to-skin contact with their infant during each hospital care day until discharge. Statistical analyses were done using a linear model with covariates.

**Results:**

Diaries were kept before and after the intervention by a total of 170 and 129 mothers and 126 and 84 fathers, respectively. Either parent was present on average 453 min per day before the intervention and 620 min after the intervention in the neonatal unit. In the adjusted model, the increase was 99 min per day (*p* = 0.0007). The infants were in skin-to-skin contact on average 76 min per day before the intervention and 114 min after the intervention. In the adjusted model, skin-to-skin contact increased by 24 min per day (*p* = 0.0405).

**Conclusion:**

The Close Collaboration with Parents intervention increased parents’ presence and skin-to-skin contact in nine hospitals. This study suggests that parent-infant closeness may be one mediating factor explaining benefits of parenting interventions.

**Trial registration:**

ClinicalTrials.govNCT04635150. Retrospectively registered.

## Background

Physical and emotional parent-infant closeness is important for the development of preterm and full-term infants [1]. Parent-infant separation during newborn care may lead to parental stress and depression and compromise parenting [2–6]. The effects of separation may be mediating factors for later behavioral problems in preterm infants [7, 8]. It is shown that parents’ involvement in infants’ care in hospital enhance long-term cognitive and neurobehavioral development of preterm infants [9–14]. Moreover, increased parental presence and availability of family rooms shorten infants’ hospital stays [15, 16]. Skin-to-Skin Contact (SSC) is an effective way to increase closeness. It reduces feelings of stress, strengthens bonding and supports the transition into new parental roles [17]. A large meta-analysis has shown that SSC reduces mortality, infections, and hospital readmissions, increases the volumes of expressed milk and the duration of breastfeeding, and improves head growth [1].

We know that a parent’s presence and SSC are safe, simple, and effective practices, but difficult to implement in neonatal environments. The difficulty of including the parents in everyday newborn care is reflected in the large variation in the amount of parent-infant closeness in different neonatal units across Europe [18]. This study aims to fill the knowledge gap related to the facilitation of parent-infant closeness. Parent-infant closeness may be supported by developing the collaborative skills of neonatal staff. It has been shown that the parents’ trustful relationship with staff decreases reported stress and supports participation in infant care [19–21]. The Close Collaboration with Parents intervention aims to improve the skills of neonatal staff for active listening and joint observations of infant behavior and collaboration with parents [22]. Staff reported after the intervention that trust increased between staff and parents and parents were more committed to infant care [23]. A more meaningful role for parents may motivate them to stay longer in the unit. Skills of the staff to observe behavior of infants in collaboration with parents and listen to the preferences of parents in a dialogue might support SSC. Parent-infant closeness may lie behind lower depressive symptoms which mothers have reported after the Close Collaboration with Parents intervention as compared to the mothers graduating from same unit before the intervention [24].

The aim of this study was to evaluate the effects of an educational intervention for neonatal staff on parent-infant physical closeness during their infant’s stay in the Neonatal Intensive Care Unit (NICU). We hypothesized that parents spend more time in the unit and have their infants more in SSC after the intervention compared to the time before the intervention.

## Methods

### Study NICUs and participants

**Fig. 1:**
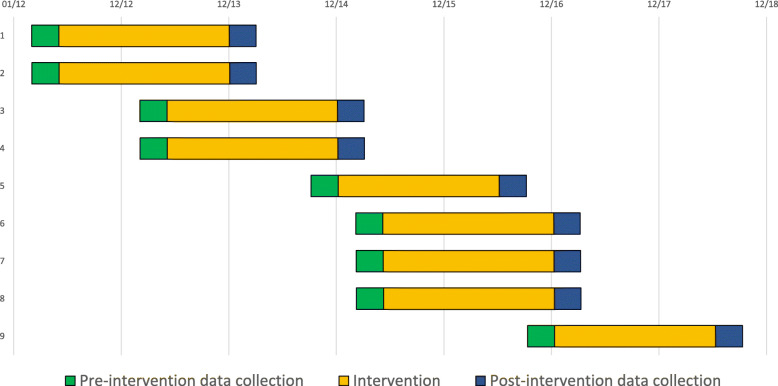
The timeline of the study process

This study was an experimental study, comparing the situation before and after the intervention. This intervention study was carried out in nine NICUs in Finland, including two level III hospitals and seven level II hospitals. The study progressed stepwise between May 2012 and September 2018; two or three hospitals participated in the study simultaneously. The data were collected in 3-month periods before and after the intervention (Fig. 1). There were no major changes in the architectural layout in the hospitals during the study period; one of the hospitals had single family rooms.

The study participants were recruited during three-month periods before and after the Close Collaboration with Parents training program, which lasted for 18 months. Thereby, the before and after samples were recruited two years apart and were independent samples. Every parent of an infant estimated to stay in the NICU longer than three days was approached. Other inclusion criteria were: 1) the infant had no major congenital anomalies or syndromes, 2) the parents spoke Finnish or Swedish, 3) the family lived in the catchment area of the hospital. A log including infant’s gestational age, birth weight, the length of hospital period and the distance to home was kept for all admissions of infants with a length of stay longer than 3 days to identify eligible parents and evaluate drop-out rate.

The staff introduced the study protocol to parents and gave them at least one day to consider their participation. After the parents signed the informed consent form, they were given instructions for use of the closeness diaries. The parents provided infant characteristics (including gestational age, birthweight, birth head circumference, sex, mode of delivery, and whether the infant was a singleton/multiple or had siblings) and family characteristics (including parents’ age, education, socio-economic status and the distance from the hospital to home).

### Closeness diary

The duration of parents’ presence in the NICU and SSC was reported with closeness diaries. Presence in the unit was defined by being inside the unit, not necessarily all the time in the room of the baby. SSC was defined as the baby lying on the parent’s bare chest dressed only in a diaper and a cap if necessary. On the diary, there were four different timelines where parents filled in the time spent in NICU and SSC with their infant: mother present, mother SSC, father present, and father SSC. Parents were asked to fill in the diaries from the time of recruitment until discharge. During data collection, the diaries were stored in a folder at the bedside so that other families or nurses did not see the diaries.

### Intervention

**Fig. 2:**
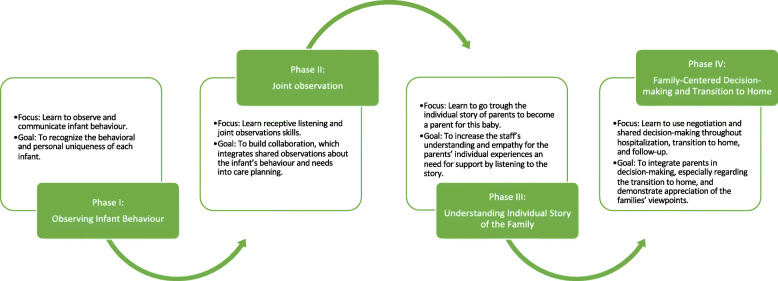
Intervention

The Close Collaboration with Parents intervention was developed based on theoretical evidence from infant neurobehavioral and attachment theories. The training is based on a multi-method learning philosophy using theoretical teaching, hands-on teaching at bedside, and reflective discussions supporting simultaneous implementation of practice change. The intervention teaches new skills to the entire staff of a unit to collaborate with parents in order to support parents’ presence and involvement in infant’s care. The original intervention was condensed to a structured 18-month-long training including four phases (Fig. 2.) [22, 25]. The content and implementation strategies of the intervention are described in the Appendix. A facilitator network model [26] was used including local mentors trained by the trainer mentors and a supervisor.

### Statistical methods

Parents’ presence was primarily approached from the infant’s perspective, defined as at least one parent present. Parents’ SSC was defined as SSC with either parent. Mothers’ and fathers’ presence and SSC were also analysed separately. We compared the pre-intervention and post-intervention cohorts adjusting for gestational age, siblings in the family, and the neonatal unit in the statistical model. These confounders were chosen based their significance on parents’ presence. The comparison of presence and SSC was analyzed using a linear model, where cohorts were independent families, siblings in the family and neonatal unit were handled as categorical variables, and gestational age as a continuous covariate. Analyses were performed for mother and father separately and then also as combined (at least one parent present, either parent SSC). All diaries until the last one were included in the analyses, also the days without presence or SSC or missing data. All statistical tests were performed as two-sided, with a significance level set at 0.05. The analyses were performed using SAS System, version 9.4 for Windows (SAS Institute Inc., Cary, NC, USA).

## Results

**Fig. 3:**
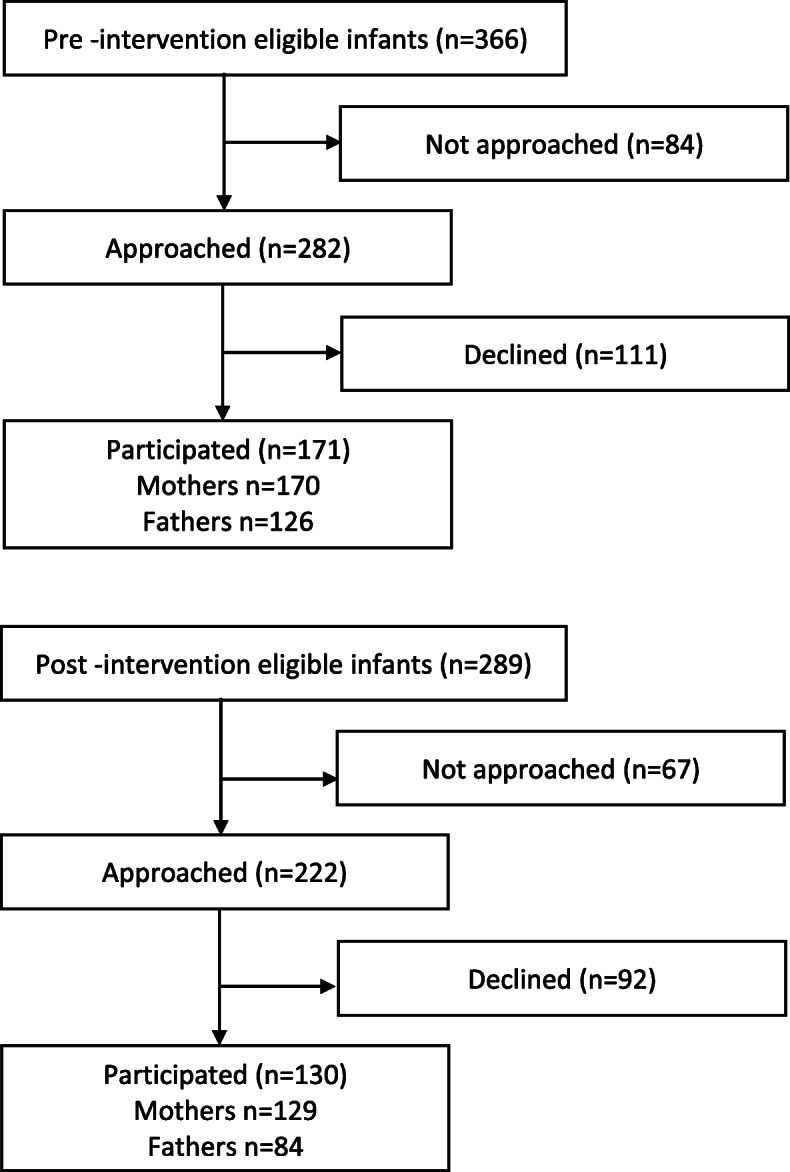
Flow chart

There were 366 eligible families before the intervention and 289 families after the intervention: 84 of the eligible families were not approached in the pre-intervention cohort and 67 in the post-intervention cohort. In the final study group, there were 171 and 130 infants (Fig. 3.). The data required for drop-out analyses were available from six out of nine study hospitals. Non-participants had higher gestational age than participants (an 11-day difference in the pre-intervention cohort; an 8-day difference in the post-intervention cohort) and birth weight (350 g and 150 g, respectively) both before and after the intervention. Closeness diaries were kept before and after the intervention by 170 and 129 mothers and 126 and 84 fathers, respectively, during their stay in the NICU. During the pre-intervention period, the mothers kept the diary for an average of 14.6 days (SD 17.3) and the fathers for 11.7 (15.0) days. During the post-intervention period, the mothers kept the diary an average of 12.7 days (SD 14.0) and the fathers for 10.2 (11.4) days. The diary days covered the majority of the hospital days from the time of recruitment until discharge.

Background factors in the pre-intervention and post-intervention cohorts did not differ significantly. The study infants were born on average 34^5/7^ (SD 4^4/7^) weeks of gestation (range 23^5/7^ to 42^0/7^) during the pre-intervention cohort and on 34 ^2/7^ (SD 4^5/7^) weeks of gestation (range 25^2/7^ to 42^0/7^) during the post-intervention cohort. In the pre- and post-intervention cohorts, 58 and 61% of the families had older siblings, respectively (Table 1.).

**Table 1:** Infant (*n* = 301) and family (*n* = 299 mothers and 210 fathers) characteristics, pre -intervention and post -intervention

Characteristics	Pre –intervention mean (SD)	Post –intervention mean (SD)
Infant	*n* = 171	*n* = 130
Gestational age	34.3 (4.5)	34.0 (4.8)
< 32	48	37
32–36	65	55
> 37	58	38
Birth weight (g)	2459 (1095)	2478 (1071)
Mode of delivery	83/86/2	60/67/3
(Ceasarean/vaginal/missing)		
Family		
Distance from home (km)	57 (80)	44 (67)
Mothers’ age	30.4 (5.1)	31.0 (5.5)
Fathers’ age	33.0 (6.3)	33.3 (6.5)
Mothers’ education		
• 9 years	0	3
• 9–12 years	40	39
• > 12 years	49	66
• Missing	81	21
Fathers’ education		
• 9 years	1	0
• 9–12 years	34	31
• > 12 years	24	36
• Missing	67	17
Couples/Single mothers/missing	166/2/3	122/5/3
Older siblings (yes/no/missing)	93/75/3	77/50/3
Closeness diary		
Days		
• Mothers	14.6 (17.3)	12.7 (14.3)
• Fathers	11.7 (15.0)	10.2 (11.4)

Parents’ presence increased from a mean of 453 min (SD 331) to 620 min (SD 371) per day after the intervention. There were differences in parents’ presence between units before the intervention. However, the effect size was similar regardless of the baseline level of the unit. In the adjusted statistical model, parents’ presence increased by 99 min after the intervention, *p* = 0.0007. Mothers’ presence was longer than fathers’ presence before the intervention, and it also increased more than fathers’ presence after the intervention. In the adjusted statistical model, mothers’ presence increased from 517 min to 624 min, *p* = 0.0004, and fathers’ presence increased from 305 min to 374 min, *p* = 0.0156 (Table 2.). Lower gestational age associated significantly with shorter parents’ presence: one day increase in gestational age corresponded with 1.4-min increase in presence, *p* = 0.0023. Having an older sibling in the family associated with shorter fathers’ presence. Fathers without older children were in the unit 394 min (SE 20.9) compared to fathers with older children were in the unit 284 min (SE 20.2), *p* < 0.0001.

**Table 2:** Parents’ presence and skin-to-skin contact in NICUs presented in minutes

Closeness	Δ mean	Lower 95% CL	Upper 95% CL	*P*-value*
Either parent present	99	42	156	0.000.7
Either parent in SSC	24	1.1	47	0.0405
Mother present	107	48	165	0.0004
Mother SSC	22	1.9	41	0.0318
Father present	68	13	124	0.0156
Father SSC	7.8	−8.8	24	0.3517

SSC, skin-to-skin contact

*adjusted by hospital, gestational age and older siblings (yes/no) in the statistical model

SSC increased from a mean of 76 min (SD 84) per day before the intervention to 114 min (SD 83) per day after the intervention. In the adjusted statistical model, parent-infant SSC increased by 24 min, *p* = 0.0405 (Table 2.). There were differences in SSC between units before the intervention, but the effect size was similar regardless of the baseline level. Mothers’ SSC was longer than fathers’ SCC before the intervention and increased more than in fathers’ SSC after the intervention. In the adjusted statistical model, mothers’ mean SSC increased from 69 min to 91 min, *p* = 0.0318 and father’s mean SSC increased from 38 min to 46 min per day, *p* = 0.3517. The proportion of days without SSC out of the days either parent was present decreased from 72 to 61% for the mothers (*p* = 0.04747) and 82 to 71% for the fathers (*p* = 0.0051). Lower gestational age associated significantly with longer SSC. A one day increase in gestational age corresponded with 0.52-min decrease in SSC, *p* = 0.0134.

## Discussion

The Close Collaboration with Parents intervention aimed to improve the skills of neonatal staff to collaborate with parents and was found to substantially increase parents’ presence and SSC in nine Finnish hospitals. We emphasized the infant perspective in the analyses by showing the time either parent was present (together or alone) in the neonatal unit. Our results showed the intervention increased parental presence by 37% and SSC by 51%.

Importantly, the intervention increased parental presence at both ends of the variation: the shortest pre-intervention presence in one of the study units was 4.2 h, which aligns with some previous studies [18, 26–28]; the highest presence was 18.7 h in a unit which had single family rooms. Parent-infant SSC varied between an average of 36 min to 182 min per day in the baseline measurements of the units. Previous studies show that the duration of SSC varies largely when comparing NICUs internationally, with some units having just less than half an hour of SSC per day while others had over 8h per day [18]. It has been shown that providing the possibility for overnight stays in the NICU increases both mothers’ and fathers’ presence and SSC [29]. The level of the NICU where the infant was born did not affect the closeness between preterm infants and their parents, suggesting that the training program can be utilized in NICUs with different acuity levels.

Earlier studies have traditionally focused on mothers’ presence only. In our study, mothers’ presence increased by 41% and fathers’ presence increased by 22%. The weaker response in the duration of fathers’ presence might reflect traditional values related to their role as a parent, conflicting needs from household work and employment, and lack of support [30]. If there were older siblings in the family, the duration of fathers’ presence was shorter than in families without siblings. Previous literature has also recognized older siblings as a barrier for parents’ presence in the NICU, especially for fathers [27, 31]. Importantly, fathers have reported that they expect the staff to invite them to be actively involved, and mixed messages about their involvement from the staff serve as primary barriers [30].

Mothers’ SSC increased by 38%, but there was no statistically significant increase in fathers’ SSC. It is interesting to speculate why fathers’ SSC did not increase even if their presence in the unit increased. It might be that SSC has been used to promote breastfeeding, which could explain lack of increase in fathers [1] Barriers and enablers for SSC include infant’s size and age and lack of knowledge about SSC [32]. However, low gestational age was not a barrier in our study, but rather increased the duration of SSC. As reported in other studies one common barrier in SSC has been the staff’s disbelief in the importance of SSC [32]. The training program also provided staff the skills to better detect the stability and well-being of infants (training program Phase I). When noticing better stability of infants during SSC, the staff may be better motivated to facilitate SSC and overcome the possible barriers. Staff also learned to notice the benefits of SSC for parents (training program Phases II and III), further supporting its implementation. As it is often more challenging to have fathers involved in SSC, fathers’ experiences related to SSC should be better understood to know how the training program could better meet their needs. The training program should emphasize the fact that SSC given by mothers and fathers is equally beneficial for the child [33], and both parents report SSC as meaningful for them [34, 35].

Our results indicate the importance of a systematic, goal-oriented approach in staff training to integrate parents in infants’ care. Our results suggest that Close Collaboration with Parents intervention promotes parental presence and SSC which has been shown to associate with better child outcomes [1, 9–14]. It is likely that traditional hospital practices do not change easily by themselves. The aim of our training program was to actively involve parents and negotiate with them about their presence and participation during newborn care. The intervention had features which enabled its adaptability in different contexts. Most importantly, the staff of the target hospitals decided themselves which practice changes were most relevant to carry out in their hospitals; the intervention aimed to change experiences, attitudes, and values behind these family centered care practices. However, this study was carried out in a high-income country that offers financial compensation for parental leaves for both parents after a delivery. Therefore, the results might not be generalizable to less affluent countries.

We changed care culture, focused on better communication with parents, and integrated parents as primary caregivers in NICUs [23]. The Close Collaboration with Parents training has been shown to decrease mothers’ depressive symptoms [36]. There have also been other parenting interventions in NICUs, but they have not measured parents’ presence [37–41]. One intervention asking parents to be present for 6 h a day improved growth in preterm infants and decreased parents’ stress and anxiety [37]. Parenting interventions have been shown to lead to better child development and increased parent wellbeing [36, 42]. The mechanisms for these positive outcomes might include psychosocial support, parenting education, developmental support and preparing parents to parenthood [43]. Our study suggests that parent-infant closeness is a potential mediating mechanism explaining the beneficial effects of parenting interventions. Therefore, it is important to include parent-infant closeness measures in parenting intervention studies.

This study did not have concurrent controls without intervention. There might be a time trend towards better understanding of the value of family centered care and, thereby, increasing in parents’ involvement during the study period. However, we did not find any time trends as parents’ presence before the intervention did not systematically increase over the study period. We wanted to implement the intervention in the whole unit in order to change the care culture of the unit. Therefore, a randomized study design within a unit was not applicable. In future, a cluster randomized study design would provide more solid information. We did not perform power calculations as we did not have preliminary information on effect size, but the significant effect proves sufficient power. The units had comparable architectural layout before and after the intervention measurements; none of them had renovations between the two measurement periods. As all nine hospitals were in Finland, this data did not prove that the training effects are similar in other countries or health care systems. However, the training program was effective in different contexts within Finland. One limitation in our study is a potential selection bias, as those parents who are less present might be less likely to be recruited for the study. However, the proportion of parents participating was similar, and high, in both before and after the intervention, so this bias is likely to have a similar effect in each cohort. In addition, we interpreted the days with empty diaries to be indicative of zero parental presence. We aimed to capture the parent perspective and collected the data from parents. Although we validated the parent diaries against nursing charts, it is possible that there are omissions in parents’ diaries [44].

## Conclusion

In summary, this study showed that an educational intervention for neonatal staff to listen parents and collaborate with them increased parents’ presence in the unit and SSC with their infant. Parent-infants closeness is likely to be an important mediating mechanism for the benefits of parenting interventions. Consistent family centered care culture, so that the entire staff works in partnership with parents, is likely to promote parents’ presence and, thereby, create better developmental environment for preterm infants. Systematic training of professionals enhances family centered care culture.

## Data Availability

The datasets generated and analyzed during the current study are not publicly available due to the terms of consent to which the participants agreed, but data are available upon reasonable request from the corresponding author.

## Abbreviations

NICU: Neonatal Intensive Care Unit
SCC: Skin-to-Skin Contact
FCC: Family Centered Care
